# Understanding dynamics of polarization via multiagent social simulation

**DOI:** 10.1007/s00146-022-01626-5

**Published:** 2023-01-21

**Authors:** Amanul Haque, Nirav Ajmeri, Munindar P. Singh

**Affiliations:** 1 Department of Computer Science, North Carolina State University, Raleigh, NC USA; 2 Department of Computer Science, University of Bristol, Bristol, UK

**Keywords:** Echo chambers, Selective exposure, User tolerance, Social networks

## Abstract

It is widely recognized that the Web contributes to user polarization, and such polarization affects not just politics but also peoples’ stances about public health, such as vaccination. Understanding polarization in social networks is challenging because it depends not only on user attitudes but also their interactions and exposure to information. We adopt Social Judgment Theory to operationalize attitude shift and model user behavior based on empirical evidence from past studies. We design a social simulation to analyze how content sharing affects user satisfaction and polarization in a social network. We investigate the influence of varying tolerance in users and selectively exposing users to congenial views. We find that (1) higher user tolerance slows down polarization and leads to lower user satisfaction; (2) higher selective exposure leads to higher polarization and lower user reach; and (3) both higher tolerance and higher selective exposure lead to a more homophilic social network.

## Introduction

As the COVID-19 pandemic crosses the 2-year mark, we can see that it has established a new normal, not only in the objective challenges it poses to society and business but also in terms of widespread attitudes and behaviors that are antivax, antimask, and antiscience. Polarization on such topics is a societal problem since it makes rational decision-making and resource allocation difficult. The Web enables fast information diffusion across traditional boundaries, which, unfortunately, has contributed to polarization. Specifically, social media influences users in subtle ways, especially regarding politics (Nahon 2015); moreover, online and offline political participation is correlated (Johnson et al. 2020; Bode et al. 2014).

We simulate two factors identified by prior research that influence polarization. First, selective exposure to congenial (attitude-conforming) information exacerbates confirmation bias, polarizing opinions further (Stroud 2010; Garrett et al. 2014; Kim 2015; Westerwick et al. 2017). Selective exposure arises in and strengthens echo chambers, wherein a person encounters only beliefs or opinions that coincide with their own so that their existing views are reinforced and alternative ideas are suppressed. Conversely, cross-cutting exposure (to uncongenial, i.e., attitude-disconfirming information) has a depolarizing effect (Kim 2015), though with caveats (Garrett et al. 2014; Kim 2019). Second, user tolerance for ideas that contradict their own mitigates polarization (Coscia and Rossi 2022).

We analyze the effects of selective exposure and tolerant users on polarization among users. Specifically, we investigate the following research questions.

RQ_tolerance_: Does higher tolerance among users in a social network help mitigate polarization?

RQ_exposure_: Does selective exposure to congenial information contribute to polarization?

We develop a multi-agent social simulation to investigate these research questions. To address RQ_tolerance_, we model tolerant users by having a higher tolerance level toward both opposing and congenial views. We operationalize tolerance in users using Social Judgment Theory (Sherif and Hovland 1961), which defines tolerant people as those having a wider latitude of non-commitment. For RQ_exposure_, we emulate selective exposure by filtering posts based on the receiving user’s stance toward a given issue.

For RQ_tolerance_, we find that tolerant users do mitigate polarization but achieve less user satisfaction than users with lower tolerance. Surprisingly, higher tolerance also leads to a more homophilic social network. For RQ_exposure_, we find that higher selective exposure leads to more polarization and a more homophilic social network. Higher selective exposure leads to higher aggregate user satisfaction in the social network but fewer satisfied users.

Analyzing polarization dynamics based on information sharing on social media can help us identify potential interventions. Since most content filtering (algorithmic selective exposure) in use today is based on artificial intelligence (AI), this work can help us better understand the social and political aspects of using AI. Our findings suggest avenues for further theoretical development in tandem with the consideration of interventions to reduce polarization in online social networks.

*Organization*: The rest of the paper is organized as follows: Sect. 2 describes the background and discusses related work. Section 3 explains our methodology, including definitions and the simulation design, assumptions, and limitations. Section 4 details the experimental setup, results of our experimentation, and statistical analysis of the results. Section 5 includes a discussion on results and threats to the validity of this work and concludes with future directions.

## Background and related work

The theory of cognitive dissonance (Festinger 1957) asserts that when a person is confronted with contrasting ideas, it causes psychological discomfort making that person more selective in their information consumption, potentially causing confirmation bias. Confirmation bias is the tendency of people to accept “confirming” evidence at face value while subjecting “dis-confirming” evidence to critical evaluation (Lord et al. 1979), resulting in people gravitating toward information that aligns with (confirms) their existing views. Bias exists in the selection and sharing of information, especially news (Hart et al. 2009; Knobloch-Westerwick 2014).

Selective exposure is a tendency of people to choose and spend more time on information that is consistent with their existing beliefs (Klapper 1960; Redlawsk 2002; Taber and Lodge 2006), though some prior works suggest that partisan selective exposure may be a myth (Kinder and Sears 1981; Zaller 1992). Freedman and Sears (1965) argue against voluntary selective exposure in favor of de facto selectivity. They claim that most examples of selectivity in mass communication can be attributed to complex factors, such as demography, education, social connections, and occupation, which are incidental to their supportiveness to the receiver’s existing beliefs. People prefer supportive information in some situations while dissonant information in other situations (Hargittai et al. 2008). Individuals with strong preferences are more likely to spend more time reading negative (uncongenial) information about their choice (Meffert et al. 2006), perhaps to critique it (Hargittai et al. 2008).

### Social media and politics

The number of users on social media platforms has increased rapidly over the years. Only 8% of Internet users in the US used some social networking platform in 2005 (Lenhart 2009), whereas in 2021, 69% used Facebook, and 40% used Instagram (Auxier and Anderson 2021). The use of social networking sites for political discussions has also increased over the years. Social media is now among the most common ways in which people, particularly young adults, obtain their political news (Infield 2020). A meta-analysis of 36 past studies assessing the relationship between social media use and participation in civic and political life found a positive correlation between the two, with more than 80% of the coefficients as positive (Boulianne 2015). Polarization measured based on online social interactions shows a good correlation with offline polarization (Morales et al. 2015). Adults who use social networking platforms as a political tool are more likely to participate in politics (Bode et al. 2014). This is true across various cultural and geographical boundaries, including empirical evidence from the US (Infield 2020), Pakistan (Ahmad et al. 2019), and Taiwan (Zhong et al. 2022).

Selective exposure to political information is correlated with polarizing people’s opinions to align with the values of the political party they support (Stroud 2010; Garrett et al. 2014; Kim 2015; Westerwick et al. 2017). The causal direction, i.e., whether selective exposure leads to polarization or the other way around, is less obvious (Stroud 2010). Stroud (2010) investigate the causal relationship between partisan selective exposure and polarization and find strong evidence suggesting selective exposure leads to polarization while finding limited evidence suggesting the reverse causal direction. Schkade et al. (2007) find that intragroup deliberation on social issues among like-minded people leads to more extreme and less diverse ideological beliefs, while Bail et al. (2018) observe that exposure to opposing views on social media can increase political polarization. Habitual online news users are less likely to exercise selectivity to get attitude-consistent exposure, which reduces their likelihood of participating in the political system (Knobloch-Westerwick and Johnson 2014). The longer individuals spend on attitude-consistent content associated with biased sources, the more immediate attitude reinforcement occurs, and its influence can be detected even after a couple of days of exposure (Westerwick et al. 2017).

Cross-cutting exposure refers to being exposed to oppositional viewpoints. Cross-cutting exposure in social networks fosters political tolerance and makes individuals aware of legitimate rationales for oppositional viewpoints (Mutz 2002b). Exposure to disagreeing viewpoints contributes to people’s ability to generate reasons, particularly why others might disagree with their view (Price et al. 2002). Kim and Chen (2016) find that exposure to cross-cutting perspectives result in a higher level of political engagement, though this increase may depend on the social media platform used.

Cross-cutting exposure, widely assumed to encourage an open and tolerant society, is not necessarily the environment that produces enthusiastically participatory individuals. People belonging to social networks involving greater political disagreement are less likely to participate in politics (Mutz 2002a, b). Constant exposure to disagreement may necessitate trade-offs in other social network characteristics, such as relationship intimacy and frequency of communication (Mutz 2002b). Conflict-avoiding individuals, in particular, are more likely to respond negatively to cross-cutting exposure by limiting their political participation to avoid confrontation and putting their social relationships at risk (Mutz 2002a).

Garrett et al. (2014) examine survey data following elections in the US and Israel and find consistent results despite cultural differences. Their findings suggest that pro- and counter-attitudinal information exposure has a distinct influence on perceptions of and attitudes toward members of opposing political parties.

Mutz (2002a) analyzes the consequences of cross-cutting exposure on political participation. They find that people whose social networks involve greater political disagreement are less likely to participate in politics and are more likely to hold politically ambivalent views. Though many studies have investigated polarization using empirical data from social media, a common limitation has been that past studies either look at one-time exposure or study these effects in isolation. For instance, Stroud (2007) studies the effects of selective exposure using empirical evidence but relies on data from one-time exposure and studies the immediate effects without differentiating the long-term effects. However, the evidence from past studies suggests that political participation and its effect are a long-term process that unfolds over time based on multiple exposures (Gerber et al. 2003; Valentino and Sears 1998). Further, existing research has focused chiefly on effect at an individual level, i.e., relying on self-reported data of how an individual’s stance is influenced by exposure to potentially polarizing content. However, self-reporting is susceptible to user bias and overlooks how changes in one part of the social network can influence other parts.

### Multi-agent social simulation

Many earlier models of opinion and influence propagation are based on a centralized diffusion process, overlooking the decentralized nature of information diffusion in social networks. Kempe et al. (2003) design two fundamental diffusion models for influence maximization, namely the independent cascade model (ICM) and the linear threshold model (LTM). Influence in these models is transferred through the correlation graph starting from a set of seed nodes (activated nodes). Influence decreases when hopping further away from the activated node.

Jiang et al. (2017) design a preference-aware and trust-based influence maximization model called the preference-based trust independent cascade model (PTICM) that takes into account user preferences and trust between users in computing influence propagation. Li et al. (2019) design a novel agent-based seeding algorithm for influence maximization named enhanced evolution-based backward selection that models individual user preferences and social context based on social influence and homophily. Their results suggest that individuals are influenced by their social context much more than retaining their own opinions. Though the Prior Commitment Level (PCL) of a user is an essential factor for influence propagation, users tend to revise their PCL over time.

Chen et al. (2020) propose a group polarization model based on the SIRS epidemic model and factor in the relationship strength based on the J–A (Jager and Amblard) model. They use a BA network model due to its closeness to the real-world social network structure and a Monte Carlo method to conduct simulation experiments.

Kozitsin and Chkhartishvili (2020) develop an agent-based model to explore how agents’ activity patterns affect the formation of echo chambers. They use a personalizing system algorithm to control mutual interactions among agents and decide what information the agents are exposed to. They find that the critical parameter that guides agents’ opinion dynamics is the probability of publishing a post, i.e., agents who often publish posts tend to enter echo chambers.

Hązła et al. (2019) use a geometric model of polarization and demonstrate that societal opinion polarization often arises as an unintended byproduct of influencers attempting to promote a product or an idea. Gaitonde et al. (2021) extend this work to show that the exact form of polarization in such models is quite nuanced. Even when strong polarization does not hold, weaker notions of polarization can attain nonetheless. Baumann et al. (2020) propose a radicalization model that uses a reinforcement mechanism to drive opinions to extremes starting from moderate initial conditions. They show that the transition from a global consensus to a radicalized state is mostly governed by social influence and the controversy in the topics discussed.

Wang et al. (2019) model a rumor-propagation framework based on information entropy to understand information distortion and its polarization effects in social networks. They find that mass polarization toward a positive or negative consensus occurs when a synergistic mechanism between preferential trust and polarization tendencies is sustained. The segregation of the population into groups of different polarities happens under certain conditions.

We design a multi-agent social simulation to emulate information diffusion on social networks. We model user behavior based on existing social science theories and empirical evidence from prior studies.

## Methodology

We now describe our social simulation model and agents’ interaction.

### Social simulation definitions

#### Definition 1 (Social Network)

*A* social network *is an undirected graph with nodes representing users and the links connecting the nodes representing a relationship between two users.*

A social network is represented as *G* = *(nodes, edges)*, where *nodes* = *{a*_1_*,..., a*_*n*_*}* are users and *edges* = *{(a*_1_*, a*_2_*), (a*_4_*, a*_9_*),..., (a*_*x*_*, a*_*y*_*)}* represent a direct connection between pair of users in the social network. An agent can only interact with its neighbors in the social network.

#### Definition 2 (Agent)

*An* agent *represents a user in the social network.*

Each agent is independent and has attributes defining its preferences, such as user activity and sharing preference. User activity captures how active an agent is, and sharing preference captures agents’ willingness to share a post on the social network. Both range over [0, 1] (0 represents most inactive/unwilling and 1 most active/willing). An agent is capable of taking two actions, sharing a post, and providing sanctions to received posts.

#### Definition 3 (Post)

*A* post *is a message shared by an agent with its neighbors in the social network.*

Agents in a social network interact by sharing posts that can be represented as *Post* = *(a, t, s)*, where *a* is the author, *t* is the topic mentioned in (or discussed in) the post, and *s* is the stance of the post toward the topic (continuous value in [*− *1, 1], where − 1 represents extreme opposition and 1 extreme support for the issue).

A post serves as a time step and is used to track changes in the social network over time. Updates to the social network and agent’s attributes are made after a post has completed diffusion in the social network (i.e., it has reached as many agents as possible).

#### Definition 4 (Sanction)

*A* sanction *is a reaction an agent has for a post it receives.*

Sanctioning provides a foundation for how participants in a sociotechnical system (STS) may seek to influence each other’s decision-making and steer the STS toward their preferred direction (Nardin et al. 2016). Agents provide positive sanctions to congenial posts and negative to uncongenial posts based on their stance on a given topic being discussed in the post. Sanctioning is analogous to providing likes and comments to a post and captures whether a user approves (likes) or disapproves (dislikes) the topic in a received post.

#### Definition 5 (Issue)

*An* issue *refers to the topic being discussed in a post.*

Issues are predefined, and all agents hold a stance on each issue. An agent’s stance toward an issue is represented as a continuous value between [*− *1, 1], with − 1 indicating extreme opposition, and 1 extreme support for the issue. Each agent has an overall POV (Point-of-View) that depends on its stance on various issues. The POV of an agent is computed as the mean of its stance on all issues. POV ranges between [*− *1, 1], with − 1 representing extreme support for POV-1 (< 0), 0 means neutral POV, and 1 extreme support for POV-2 (> 0).

With respect to a post, an agent can be in one of the four states: (1) Non-receiver: Agents who have not yet received the post (all agents other than the author are in this state at the start of the simulation); (2) Receiver: Agents who have received the post (but not yet shared it); (3) Spreader: Agents who have shared the post with their friends; and (4) Disinterested: Agents who received the post but chose not to share it further and lost interest in the post.

### Social simulation model

The simulation starts with an agent (${a}_{x}$) sharing a post (${p}_{k}$) with its neighbors in the social network. The receiver then decides whether to share the received post further with a probability of sharing that depends on the content of the post and the receiver’s preferences. An agents’ preference involves its sharing preference, how active the agent is on the social network, and the agent’s stance toward the issue (supporting vs. opposing). The content of a post includes the issue mentioned in the post and the post’s stance toward the issue. Equation 1 describes the computation for sharing probability $sP({a}_{x}, {p}_{k})$ for the agent ${a}_{x}$ to share the post ${p}_{k}$ it received.

1 $$sP({a}_{x}, {p}_{k}) = {c}_{1} \times uA({a}_{x}, {p}_{k-1}) \times |uS({a}_{x}, i, {p}_{k-1}) \times pS({p}_{k}, i)| \times s\mathrm{Pref}({a}_{x}, {p}_{k-1})$$

${c}_{1}$ is a constant, ${a}_{x}$ is the receiver, ${p}_{k}$ is the *k*th post being shared in the social network, and $i$ is the issue discussed in the shared post. $uA\left({a}_{x}, {p}_{k-1}\right)$ is the user activity of user ${a}_{x}$ before the post ${p}_{k}$ is shared, $uS({a}_{x}, i, {p}_{k-1})$ is the user ${a}_{x}$’s stance toward issue $i$ before the post ${p}_{k}$ is shared, $pS({p}_{k}, i)$ is the stance of the post toward issue $i$ and $s\mathrm{Pref}({a}_{x}, {p}_{k-1})$ is the sharing preference of user ${a}_{x}$ before the post ${p}_{k}$ is shared. An agent with low $s\mathrm{Pref}({a}_{x}, {p}_{k-1})$ is more likely not to share a post further and may enter the state Disinterested. Disinterested agents are not candidates for sharing the post (${p}_{k}$) further.

The agents who receive the post provide a sanction. Sanctions can be positive or negative. Sanctions by the receiver depend on how active the receiver is, its stance toward the issue at hand, and the post’s stance toward the issue. Sanction by an agent ${a}_{y}$ for a post ${p}_{k}$ it received from agent ${a}_{x}$ is computed using Eq. 2.

2 $$\mathrm{Sanc}({a}_{y}, {p}_{k}, {a}_{x}) = {c}_{1} \times uA({a}_{y}, {p}_{k-1}) \times uS({a}_{y}, i, {p}_{k-1}) \times pS({p}_{k}, i)$$

$\mathrm{Sanc}({a}_{y}, {p}_{k}, {a}_{x})$ is a sanction provided by agent ${a}_{y}$ for the post ${p}_{k}$ it received from agent ${a}_{x}$. Sanction scores affect user activity and the stance of each agent toward an issue. Agents prefer positive sanctions (social acceptance), which increase their activity on the platform, while negative sanctions discourage agents from sharing their views in future, hence reducing their participation (user activity). The update in user activity depends on the sanctions received by an agent for the posts it shared. An agent’s user activity $uA\left({a}_{x},{p}_{k}\right)$ after sharing a post ${p}_{k}$ is computed using Eq. 3.

3 $$uA\left({a}_{x},{p}_{k}\right)=uA\left({a}_{x},{p}_{k-1}\right)+ {c}_{2} \times \sum_{{a}_{i} \in \mathrm{neighbor}(G, {a}_{x}, {p}_{k})}\mathrm{Sanc}({a}_{i}, {p}_{k}, {a}_{x})$$

${c}_{2}$ is a constant, $uA\left({a}_{x},{p}_{k-1}\right)$ represents the user activity of agent ${a}_{x}$ before the post ${p}_{k}$ is shared, and $uA\left({a}_{x},{p}_{k}\right)$ represents the user activity of agent ${a}_{x}$ after the post ${p}_{k}$ is shared, $\mathrm{neighbor}\left(G, {a}_{x}, {p}_{k}\right)$ refers to all neighbors of agent ${a}_{x}$ in the social network $G$ that receive the post ${p}_{k}$ directly from agent ${a}_{x}$.

An agent’s stance toward an issue is influenced by the sanctions it receives from other agents. We model this shift in the stance of an agent using Social Judgment Theory (SJT) (Sherif and Hovland 1961), which describes how individuals change their position when confronted with another position on a given issue. According to SJT, an individual shifts their stance in the direction of the contradicting stance if the contradicting stance falls within their latitude of acceptance (assimilation). In contrast, they will shift away from the contradicting stance (i.e., bolster existing beliefs) if the contradicting stance falls beyond their latitude of rejectance (contrast). For instance, for an agent ${a}_{x}$, that has a stance of $uS({a}_{x}, i, {p}_{k})$ toward issue $i$, a threshold determining the latitude of acceptance ${u}_{xi}$ and a threshold determining the latitude of rejection ${t}_{xi}$ with ${t}_{xi}$ > ${u}_{xi}$. When this agent ${a}_{x}$ interacts with another agent ${a}_{y}$, the following rules are applied to compute the shift in the stance of agent ${a}_{x}$ toward an issue $i$.

4 $$\mathrm{diff}\_\mathrm{Stance}({a}_{x}, {a}_{y}, i, {p}_{k}) = |uS({a}_{x}, i, {p}_{k}) - uS({a}_{y}, i, {p}_{k})|$$

d$\mathrm{iff}\_\mathrm{Stance}({a}_{x}, {a}_{y}, i, {p}_{k})$ is the absolute difference in the stances of agent ${a}_{x}$ and agent ${a}_{y}$ on the issue $i$ as the post ${p}_{k}$ is being shared.

5 $$\begin{gathered} {\text{If}}\;{\text{diff}}\_{\text{Stance}}\left( {a_{x} , a_{y} , i, p_{k} } \right) < u_{xi} \quad \delta uS\left( {a_{x} , a_{y} , i, p_{k} } \right){ } = { }\mu \times \left( {uS\left( {a_{y} , i, p_{k} } \right){ } - uS\left( {a_{x} , i, p_{k} } \right)} \right) \hfill \\ {\text{If}}\;{\text{diff}}\_{\text{Stance}}\left( {a_{x} , a_{y} , i, p_{k} } \right) > t_{xi} \quad \delta uS\left( {a_{x} , a_{y} , i, p_{k} } \right){ } = { }\mu \times \left( {uS\left( {a_{x} , i, p_{k} } \right){ } - uS\left( {a_{y} , i, p_{k} } \right)} \right) \hfill \\ {\text{else}}\;\delta uS\left( {a_{x} , a_{y} , i, p_{k} } \right) = 0 \hfill \\ \end{gathered}$$

$\upmu$ represents the strength of the influence between two agents. We assume the same strength of influence between all pairs of connected agents in the social network; hence the value of $\mu$ is 1. The shift in the stance of an agent ${a}_{x}$ for sharing posts ${p}_{k}$ on issue $i$ is computed using the received sanction scores and the difference in stance (toward the issue at hand) between the author or spreader (i.e., ${a}_{x}$) of the post, and the receiver (i.e., ${a}_{y}$) (Eq. 6).

6 $${\Delta S\left({a}_{x}, {a}_{j}, i, {p}_{k}\right)=c}_{2}\times \frac{\mathrm{Sanc}({a}_{y}, { p}_{k}, { a}_{x})}{\delta uS\left({{a}_{x}, a}_{y}, { i, p}_{k}\right)+1}$$

$\Delta S({a}_{x}, {a}_{j}, i, {p}_{k})$ is the shift in stance (of agent ${a}_{x}$) due to a sanction (by agent ${a}_{y}$) for a post ${p}_{k}$ it shared on the issue $i$*.*

User stance after sharing post ${p}_{k}$ can be computed using Eq. 7.

7 $$uS\left({a}_{x}, i, {p}_{k}\right)= uS\left({a}_{x}, i, {p}_{k-1}\right)+ \sum_{{a}_{j} \in \mathrm{neighbor}(G, {a}_{x}, {p}_{k})}\Delta S({a}_{x}, {a}_{j}, i, {p}_{k})$$

$uS\left({a}_{x}, i, {p}_{k-1}\right)$ is the stance of the agent ${a}_{x}$ on issue $i$ before it shares post ${p}_{k}$, and $uS\left({a}_{x}, i, {p}_{k}\right)$ is the stance of an agent ${a}_{x}$ on issue $i$ after the posts ${p}_{k}$ is shared, and sanctions for it received from all other agents. The maximum allowed change in stance due to one post is 0.20, and we bound user stance within [−1, 1] by restricting the values.

The codebase^1^ of our social simulation is publicly available. The codebase also includes the initial seed data used in our simulation.

### Agent goals and actions

The simulation progresses with agents sharing posts with other agents, causing each post to diffuse further in the social network. Each post receives a sanction from all agents that receive it, and these sanctions, in turn, influence its authors’ (spreaders’) activity score and stance toward various issues. An agent supports a POV (Point-of-View) with which its aggregate stance toward various issues is in agreement. Agents can take two actions, sharing a post and sanctioning a received post. Agents in the simulation try to maximize their influence and popularity in the social network by sharing relevant content and providing appropriate sanctions. Accordingly, we define two goals for each agent—Promoting Views and User Satisfaction.

*Promoting views*: All agents try to promote their views (POVs) on different issues by sharing relevant posts with their friends (neighbors in the social network). Agents also achieve this by providing positive sanctions to what agrees with their views and negative sanctions to what does not.

*User satisfaction*: All agents try to maximize their satisfaction. User satisfaction is computed based on the sanctions received from other agents. Agents change their stance toward issues to ensure more aggregate positive sanctions over time.

### Simplifying assumptions

We make simplifying assumptions to operationalize user attributes and online sharing behavior.

First, we assume views (on an issue) to be binary in this simulation, i.e., either supporting POV-1 or POV-2, meaning agents with no POV are non-participating. This is a design choice as we intend to analyze the scenario where only motivated agents (i.e., agents with a POV) try to influence and promote their views. As an agent becomes neutral in its POV (i.e., an agent with POV as zero), it stops sharing posts and providing sanctions. We assume all agents have some POV at the start of the simulation, and no agent has a neutral POV.

Second, we assume the initial user attributes and stance of each post based on a probability distribution. We use a random normal distribution to populate initial user attributes, including the agent’s stance toward an issue, sharing preference, and post’s stance. This ensures a balance of stance toward each POV across issues and provides a reasonable starting condition for the simulation.

Third, we assume all agents prefer getting positive sanctions over negative or none. They accordingly change their stance on issues over time to ensure social acceptance (i.e., to get aggregate positive sanctions from their neighbors). Sanctions also influence user activity; positive sanctions cause higher user activity, while negative sanctions cause it to decline.

#### Limitations

Our simulation models user preferences and emulates user behavior on social networks to analyze polarization dynamics. However, our model has a few limitations that stem from the simplifications (of user behavior and its influence).

First, for simplicity, sharing of posts and opinion shifts are sequential in this simulation, i.e., only one post is being shared in the network at any given time. Another post starts diffusing in the network only when the previous post has completely diffused (i.e., has reached all agents it could have). This limits the simulation to not factor in the effects of parallel exposure to different (maybe conflicting) information, i.e., being exposed to several posts relating to an issue before forming (shifting) an opinion about it.

Second, the social network in this simulation is static, i.e., neither a new link is formed nor an existing one severed at any time. However, selective exposure partially makes the network dynamic by filtering posts based on the difference in stance between two agents toward an issue. A dynamic social network demands far more computational resources and some knowledge of the offline world to link or delink agents over time appropriately.

## Experiments and results

We now describe the experimental setup and the metrics used to measure changes in the social network, followed by results.

### Initial simulation setup

We use the Facebook social network from Leskovec and Mcauley (2012) to seed the simulation. The social network consists of 4039 nodes (agents) and 88,234 edges (neighbors) and an average clustering coefficient of 0.61.

The agents in the social network interact by sharing posts from a pool of artificially generated posts without replacement. The stance of the posts follows a bounded normal distribution (*µ* = 0.00, *σ* = 0.52, min =  *− *1, max = 1) such that there is equal support and opposition for each issue. We predefine six issues and generate an equal number of posts for each issue. We use a total of *≈*5,000 posts that are shared between agents in each run of the experiment. Each simulation run ends when all posts in the pool of generated posts have been shared in the social network.

We create ten independent initial distributions to assign different initial user attributes for each simulation run. We set initial user satisfaction to zero for all agents. Each agent is initialized with a sharing preference based on a random normal distribution bounded between 0 and 1 (average over all distributions, *µ* = 0.5, *σ* = 0.14, min = 0, and max = 1). User activity is initialized based on a tailed distribution bounded between 0 and 1, skewed toward higher values (average over all distributions, *µ* = 0.874, *σ* = 0.17, min = 0, and max = 1). Higher initial user activity ensures greater activity and faster results. We compute kurtosis (Zwillinger and Kokoska 1999) for all user activity distributions to measure the tail of the distributions. The average kurtosis (over all ten distributions of user activity) was 1.54 (for a normal distribution kurtosis is close to zero).

We assume two POVs (Point-Of-Views), POV-1 and POV-2. Each agent has a POV in [*− *1, 1] that depends on its stance on various issues. Each agent’s stance toward different issues is initialized based on a random normal distribution bounded in [*− *1, 1] centered around zero. The stance distribution is such that on aggregate, there is equal support and opposition for each issue. The POV of each agent is computed as the average stance on issues favoring each POV, resulting in a normal distribution in [*− *1, 1] approximately centered around zero (average over all distributions, *µ* = 0.01, *σ* = 0.11, min = − 0.40, and max = 0.44). This ensures there is approximately equal support for each POV at the start of the simulation.

We ensure consistency between the agent stance who authors and shares the post and the stance of the post by choosing the authors appropriately. If an agent supports issue A, it will only start a supportive post on issue A, whereas an agent who opposes it starts only a critical one on that issue. Agents are chosen to be authors of a post based on their activity score and sharing preference half of the time and at random for the other half. Agents who are more active or have a higher sharing preference are more likely to start sharing a post.

### Metrics

We define primary and secondary metrics to measure various changes in the network over time. Primary metrics focus on measuring polarization and user satisfaction, while secondary metrics compare initial and final user distribution for different user attributes in each experiment.

#### Primary metrics

Primary metrics include the following.

*Polarization*: Polarization measures the extent to which the resulting distribution of opinions is polarized. We adopt the polarization index measure proposed by Morales et al. (2015) to measure overall polarization in the social network. The polarization index is inspired by the electric dipole moment and measures polarization as the distance between two opposing ideologies. Polarization lies in [0, 1], with 0 indicating the least polarization and 1 indicating the most.

To compute polarization, we define *A*^*−*^ as the relative population with POV-1 (i.e., negative POV, < 0) and *A*^+^ as the relative population with POV-2 (i.e., positive POV, > 0). We compute the normalized difference in the populations using the relative populations *A*^*−*^ and *A*^+^.

8 $$\Delta A = |A^{ + } - A^{ - } |$$

We then compute the gravity center (mean) of each population, *gc*^*−*^ and *gc*^+^, and define the pole distance, *d*, as the normalized distance between the two gravity centers. *d* can be expressed as.

9 $$d= \frac{|{gc}^{+}- {gc}^{-}|}{|\mathrm{max}\left({A}^{+}\right) - \mathrm{min}({A}^{-}) |}$$

max(*A*^+^) expresses the maximum possible value for positive opinions (POV > 0), and min(*A*^*−*^) expresses the minimum possible value for negative opinions (POV < 0).

The network polarization ($\mathrm{Polarization}(G, {p}_{k})$) after the post ${p}_{k}$ is shared on the social network is defined based on the function of the difference in size between the population of both POVs (∆*A*) and the pole distance *d*.

10 $$\mathrm{Polarization}(G, {p}_{k}) = (1 - \Delta A)d$$

*Polarity*: Polarity is indicative of the POV that has greater aggregate support in the social network. We measure polarity as the mean POV of all agents. Polarity ranges over [*− *1, 1], with − 1 indicating absolute support (by all agents) for one POV (POV-1) and + 1 for the other (POV-2), and 0 for neutral POV.

11 $$\mathrm{Polarity}\left(G,{p}_{k}\right)=\sum_{{a}_{i }\in G} \frac{\mathrm{POV}({a}_{i}, {p}_{k})}{\mathrm{numAgents}(G)}$$

*Homophily*: Homophily measures the homogeneity of a network structure with respect to some attribute (i.e., the agents’ POV in this case). Homophily is shown to be useful in link prediction between users in a social network (Yuan et al. 2014). Higher homophily is indicative of greater segregation in the social network. We use the assortativity of a social network (Newman 2003) to measure homophily. The value of homophily ranges over [*− *1*,* 1], with 1 indicating a perfectly assortative network and values in [*− *1*,* 0] indicating a perfectly disassortative network.

12 $$\mathrm{Homophily}\left(G,{p}_{k}\right)= \frac{\sum_{i,j}{e}_{ij }- \sum_{i,j}{a}_{i}{b}_{i}}{1-\sum_{i,j}{a}_{i}{b}_{j}}$$

where ${e}_{ij}$ is the fraction of edges in a network that connects a vertex of type $i$ to one of type $j$, and ${a}_{i}$ and ${b}_{j}$ are the fractions of each type (based on the agents’ POV) of the end of an edge attached to vertices of type $i$, and type $j$ respectively. The type depends on the agent’s POV, and we group agents into 20 equally spaced groups based on their POV. We use the networkx^2^ implementation of assortativity to compute network homophily.

*User satisfaction*: User satisfaction measures how satisfied the overall social network is based on the outcome of individual user actions. To operationalize the computation for user satisfaction (for each agent), we use the sanction scores that an agent gets for sharing posts with other agents in the social network to compute the update in user satisfaction (Eq. 13). We take the mean of each user’s satisfaction to compute overall network satisfaction (Eq. 14).

13 $$\mathrm{uSat}\left({a}_{x}, {p}_{k}\right)=\mathrm{uSat}\left({a}_{x},{p}_{k-1}\right)+{c}_{2} \times \sum_{{a}_{i} \in \mathrm{neighbor}(G,{a}_{x},{p}_{k})}\mathrm{Sanc}({a}_{i}, {p}_{k}, {a}_{x})$$

14 $$\mathrm{netSat}\left(G,{p}_{k}\right)= \sum_{{a}_{i} \in G}\frac{\mathrm{uSat}({a}_{i},{p}_{k})}{\mathrm{numAgent}(G)}$$

where $\mathrm{uSat}\left({a}_{x}, {p}_{k}\right)$ refers to the user satisfaction of agent ${a}_{x}$ after the post ${p}_{k}$ has been shared, $\mathrm{uSat}\left({a}_{x},{p}_{k-1}\right)$ refers to the user satisfaction of agent ${a}_{x}$ before the post ${p}_{k}$ has been shared, and $\mathrm{netSat}\left(G,{p}_{k}\right)$ measures the overall network user satisfaction after post ${p}_{k}$ has been shared.

#### Secondary metrics

We define secondary metrics to compare user distribution (based on count) in the initial (at the start of the simulation run) and final (after completion of each simulation run) populations. We define three secondary metrics based on user attributes (such as user activity and user’s POV), and the primary metric on user satisfaction. Secondary metrics are computed after all posts are shared. Secondary metrics include the following:

*Satisfied users*: User distribution (percentage) in initial and final populations with negative (< 0), zero (= 0), or positive (> 0) user satisfaction.

*Active users*: User distribution (percentage) in initial and final populations with low (< 0.75), medium (> 0.75 and < 0.90), or high (> 0.90) user activity.

*Polarized users*: User distribution (percentage) in initial and final populations with high (> 0.10 or <  *− *0*.*10) or low (> *− *0*.*10 and < 0.10) intensity of POVs.

Table A.2 describes the secondary metrics and lists their thresholds.

### Experiments

To address RQ_tolerance_ (Does higher tolerance among users in a social network help mitigate polarization?), we vary agents’ tolerance levels. To address RQ_exposure_ (Does selective exposure to congenial information contribute to polarization?), we vary the levels of selective exposure in our simulation. We analyze the influence of changing these configurations on the primary and secondary metrics.

To mitigate the effects of stochasticity, we run the simulation ten times with different initial distributions for the agent’s attributes while keeping the social network and shared posts the same to ensure a fair comparison. For each experiment, we compute the primary and secondary metrics. The reported results are averages of ten simulation runs.

**Fig. 1:**
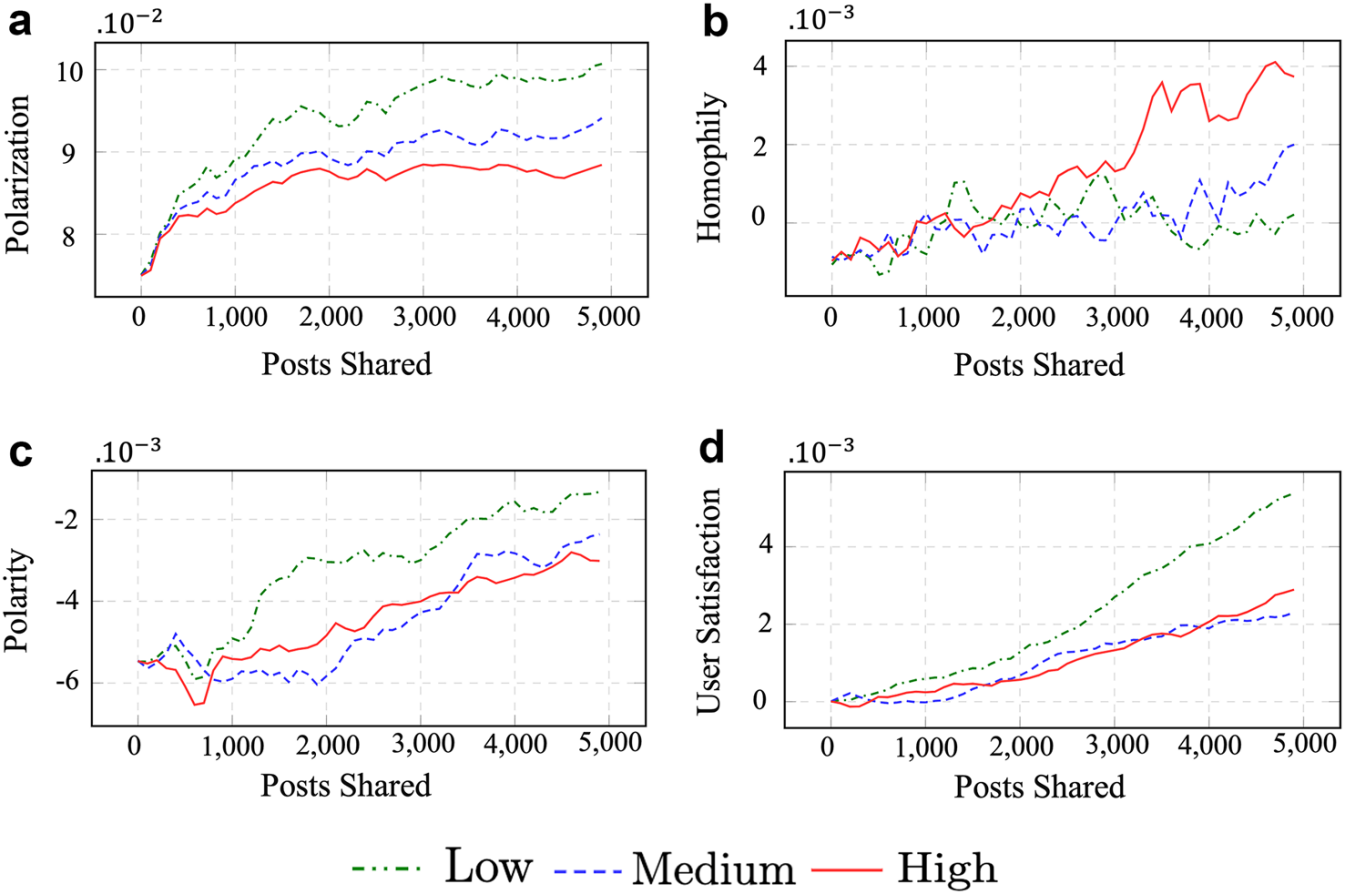
Experiment 1 (tolerance): comparing polarization, homophily, network polarity, and user satisfaction of agents in a social network with different tolerance levels

**Fig. 2:**
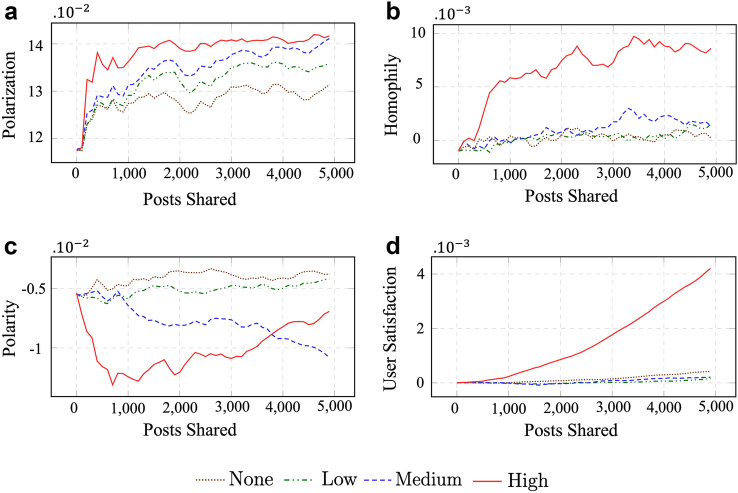
Experiment 2 (selective exposure): comparing polarization, homophily, polarity, and user satisfaction of agents in a social network with different levels of selective exposure

Figures 1 and 2 compare how polarization, polarity, homophily, and user satisfaction change with more posts being shared under different experimental settings. Tables 1 and 2 summarize our findings for the two experiments. Tables 4 and 5 include results from the statistical analysis. Tables A.1 and A.2 include a description of the notations used to explain the simulation design and metrics, respectively. Sections 4.3.1 and 4.3.2 describe the experimental setup and results of the two experiments in detail.

**Table 1:** Distribution of agents across different states in the final population for each experimental setting

Exp	Config	Agent state		
		Non-receiver	Receiver	
			Spreader	Disinterested
Tolerant users	Low	60*.*12	14*.*49	25*.*39
	Medium	53*.*95	17*.*30	28*.*75
	High	62*.*99	13*.*36	23*.*65
Selective exposure	None	54*.*76	16*.*88	28*.*36
	Low	55*.*44	16*.*48	28*.*08
	Medium	58*.*90	13*.*80	27*.*30
	High	82*.*63	4*.*97	12*.*40

Results are from averages of ten simulation runs. Values are in % of the total population

**Table 2:** Comparison between initial and final distributions of agents based on secondary metrics for different experiments

Exp	Conf	User Satisfaction			User Activity			User Polarity	
Initial distribution		Neg	Zero	Pos	Low	Med	High	Low	High
		0.00	100.0	0.00	1.56	64.82	33.62	99.33	0.67
Tolerant users	Low	52*.*09	23*.*79	24*.*11	4*.*51	64*.*79	30*.*70	97*.*50	2*.*50
	Medium	51*.*92	22*.*26	25*.*82	4*.*51	64*.*52	30*.*97	98*.*19	1*.*81
	High	50*.*11	23*.*42	26*.*47	4*.*33	65*.*39	30*.*28	98*.*54	1*.*46
Selective exposure	None	51*.*74	23*.*25	24*.*01	4*.*48	64*.*82	30*.*70	98*.*69	1*.*31
	Low	51*.*40	22*.*93	25*.*67	5*.*08	64*.*62	30*.*30	98*.*69	1*.*31
	Medium	45*.*26	26*.*64	28*.*10	7*.*30	64*.*42	28*.*28	97*.*03	2*.*97
	High	23*.*75	53*.*97	22*.*28	4*.*35	63*.*98	31*.*67	96*.*73	3*.*27

Results are from averages of ten simulation runs. Values are in % of the total population

#### Experiment 1: tolerant users

The tolerance of an agent is defined based on its latitude of non-commitment (Sherif and Hovland 1961), i.e., the difference between the latitude of acceptance (assimilation) and latitude of rejectance (contrast). The higher difference implies more tolerance. A more tolerant agent is less reactive to sanctions it receives from other agents for its shared posts, i.e., a more tolerant agent is less likely to change its stance on issues based on sanctions from agents who differ from its stance above a threshold (level of tolerance).

We run the simulation with three levels of tolerance, namely, High, Medium, and Low. High tolerant agents have a higher latitude of non-commitment (70%) and change their stance only based on sanctions from agents within a smaller (30%) difference in stance (between receiver and spreader) toward an issue. If a High tolerant agent receives a sanction from an agent who differs in stance (on the issue in the shared post) by greater than 30%, it discards that sanction and does not update its stance. Medium tolerant agents have a latitude of non-commitment of 40%, and low tolerant agents have a latitude of non-commitment of 10%.

Figure 1 shows changes in the primary metrics as more and more posts are shared. When agents have a High tolerance, polarization grows slower than when tolerance is Medium or Low. The polarization is constantly lower when tolerance in agents is High compared to Medium or Low. Homophily grows faster when the agent’s tolerance is High, compared to Medium or Low, and social networks whose agents have higher tolerance end up with higher homophily after all posts are shared. The overall user satisfaction at Low tolerance is constantly higher than High or Medium.

Table 1 shows the proportion of receiver (spreader and disinterested) and non-receiver agents after all the posts are shared. The number of receivers (agents who receive a post) is the highest when tolerance is Medium and the lowest when tolerance is High. The number of disinterested agents is the highest when tolerance is High.

Table 2 lists values for secondary metrics after all posts are shared. Secondary metrics compare the proportion of satisfied, active, and polarized users in the initial (before any posts are shared) and final (after sharing 5000 posts) populations based on thresholds defined for secondary metrics (Table 2). The number of positively satisfied users is the highest when tolerance in users is High and the lowest when tolerance is Low. User activity shows minor variation across different levels of tolerance. Low tolerance leads to the highest increase in highly polarized agents, whereas it is the lowest when tolerance in agents is High.

**Table:** 

Takeaway (tolerance)
Higher tolerance in users slows down polarization leading to a less polarized network, higher network homophily, lower user satisfaction, and a low number of highly polarized users than when tolerance in users is lower

#### Experiment 2: selective exposure

We emulate selective exposure in our simulation by exposing each agent only to posts from other agents who have a similar stance on the issue discussed in the post. To operationalize selective exposure, we use a threshold value of the difference in the stances of two agents beyond which they stop seeing each other’s posts. An agent sees posts only from other agents whose stance differs from its stance on an issue in the post below a threshold.

We experiment with four threshold values for selective exposure, None (allow all agents to see all content shared by neighbors without any filtering, i.e., no selective exposure), Low (allow a difference of 80% in the stance between sharing and receiving agents toward the issue in the post), Medium (allow 50% difference), and High (allow 20% difference). We maintain the tolerance level among users at Medium for all scenarios in this experiment.

Figure 2 compares the influence of different levels of selective exposure on all primary metrics. High selective exposure leads to the highest polarization, and None leads to the lowest. Polarization in a social network is constantly higher for higher levels of selective exposure. Homophily is the highest when selective exposure is High, and shows minor variations across lowers levels of selective exposure. User satisfaction is the highest when selective exposure is High and shows minor differences across lower levels of selective exposure.

Table 1 shows the proportion of receiver (spreader and disinterested) and non-receiver agents after all posts are shared. High selective exposure experiences the lowest proportion of receiver agents, while None selective exposure leads to the most.

Table 2 compares the proportion of satisfied, active, and polarized users in the initial (before any posts are shared) and final (after sharing 5000 posts) populations based on thresholds defined for secondary metrics (Table 2). Medium selective exposure experiences the highest number of positively satisfied users, whereas the highest number of negatively satisfied users is with None selective exposure. High selective exposure leads to the lowest number of negatively satisfied users. The number of highly active users experiences the most decline when selective exposure is Medium, and the least when selective exposure is High. High selective exposure leads to the highest number of highly polarized users, whereas None and Low selective exposure lead to the lowest.

**Table:** 

Takeaway (selective exposure)
Higher selective exposure leads to higher polarization, higher network homophily, higher overall user satisfaction, and a higher number of polarized users than when selective exposure is lower

#### Statistical analysis

We conduct statistical analysis to test if different levels of selective exposure and tolerance lead to statistically significant differences in users’ POV (point-of-view) and primary metrics (network polarization, homophily, polarity, and user satisfaction). For users’ POV we compare the final distributions (after all posts are shared) of users’ POV at different levels of selective exposure and tolerance to establish if the differences are statistically significant. For primary metrics, we compare the distributions of each primary metric (computed after sharing each post) at different levels of selective exposure and tolerance to identify differences in the overall social network metrics.

To choose the applicable statistical tests appropriately, we first evaluate the distributions. We test the normality of distribution using the Shapiro–Wilk normality test (Shapiro and Wilk 1965). We use parametric statistical tests, namely paired *t* test and one-way ANOVA, to compare normal distributions, and non-parametric tests, namely the Kruskal–Wallis test, for distributions that are not normal.

In addition to the statistical significance test, we also compute the effect size for each test. For parametric statistical tests we use Cohen’s *d* (Cohen 1988) to compute the effect size as the distributions under comparison have similar standard deviations and the sample size is large (≈ 4000). To interpret the effect size computed using Cohen’s *d* we adapt the interpretation from Cohen (1988) (see Table 3). For nonparametric statistical tests (Kruskal–Wallis test), we use epsilon square (${\varepsilon }^{2}$) (Kelley 1935) to compute the effect size based on recommendations from Tomczak and Tomczak (2014). To interpret the effect size computed using epsilon square (${\varepsilon }^{2}$), we adapt the interpretation from Rea and Parker (2014) for the correlation coefficient and square the threshold values of each bin as ${\varepsilon }^{2}$ is a squared metric. The resulting interpretation for ${\varepsilon }^{2}$ effect size we use is as shown in Table 3. We chose ${\varepsilon }^{2}$ over other popular alternatives, such as omega-squared (ω^2^) (Albers and Lakens 2018), as ${\varepsilon }^{2}$ is less biased (Okada 2013).

**Table 3:** Effect size and their interpretations

Metric	Effect size	Interpretation
Epsilon-square (${\varepsilon }^{2}$) Interpretation based on (Rea and Parker 2014)	[0.00, 0.01)	Negligible
	[0.01, 0.04)	Weak
	[0.04, 0.16)	Moderate
	[0.16, 0.36)	Relatively strong
	[0.36, 0.64)	Strong
	[0.64, 1.00]	Very strong
Cohens’ *d* Interpretation based on (Cohen 1988)	0.20	Small
	0.50	Medium
	0.80	Large

For all statistical significance tests, we assume the null hypothesis to indicate similar distribution  while the alternate hypothesis to indicate that there exist statistically significant differences in the compared distributions. We use the significance level, i.e., alpha, as 0.05 to accept or reject the null hypothesis.

We use the Kruskal–Wallis test to compare all primary metrics for different levels of selective exposure and user tolerance. For selective exposure, we compare how different levels (i.e., Low, Medium, and High) compare against None selective exposure, whereas for user tolerance, we compare each level of tolerance against each other in pairs.

Table 4 shows the results of the statistical significance test for all primary metrics at different levels of selective exposure and tolerance. The compared distributions correspond to the value of each metric after each post is shared on the social network. We are effectively comparing how the social network evolves (in terms of the metrics) as more and more posts are shared. The *p* values for each pair of distributions comparing the metrics indicate that the difference in the distributions is statistically significant, and the null hypothesis can be rejected, though the effect sizes vary. Based on the effect size, the difference between network homophily when selective exposure is Medium and High (compared to None selective exposure) is very strong. The difference in polarization at High selective exposure (compared with None) and High tolerance (compared with Low) is strong. Similarly, the difference in homophily between Low and None selective exposure and user satisfaction between High and None selective exposure is also strong. For different levels of user tolerance, relatively strong differences exist in polarization between Low and Medium, Medium and High; in homophily between Low and Medium, High and Low; and in polarity between High and Low. For different levels of selective exposure, a relatively strong difference (in comparison to None selective exposure) exists in polarization at High; in polarity at Medium; and in user satisfaction at Low. Other comparisons have an effect size of either moderate or weak. Table 5 shows the results of the statistical significance test comparing users’ POV at different levels of selective exposure and tolerance. The compared distributions correspond to the POV of each user after all posts are shared on the social network. We are effectively comparing how the POV of users differ as a consequence of different levels of selective exposure and tolerance at the start and end of each simulation run. The *p* values for some of the differences show that the differences are statistically significant, though the effect sizes are either small or very small.

**Table 4:** Statistical significance test results comparing primary metrics across different levels of selective exposure and user tolerance

Exp	Metric	Dist1	Dist2	*H* statistic	*p* value	Effect size
Tolerant users	Polarization	Low	Medium	2784*.*62	< 0.01	0.27
		Medium	High	2852*.*45	< 0.01	0.28
		High	Low	4178*.*42	< 0.01	0.42
	Homophily	Low	Medium	1894*.*71	< 0.01	0.19
		Medium	High	15*.*27	< 0.01	0.00
		High	Low	2353*.*32	< 0.01	0.24
	Polarity	Low	Medium	67*.*88	< 0.01	0.01
		Medium	High	1516*.*77	< 0.01	0.15
		High	Low	1981*.*18	< 0.01	0.20
	User Satisfaction	Low	Medium	1111*.*50	< 0.01	0.11
		Medium	High	10*.*60	< 0.01	0.00
		High	Low	1075*.*30	< 0.01	0.11
Selective exposure	Polarization	None	Low	1336*.*62	< 0.01	0.13
		None	Medium	2918*.*22	< 0.01	0.29
		None	High	4317*.*15	< 0.01	0.43
	Homophily	None	Low	5038*.*38	= 0.04	0.50
		None	Medium	7316*.*85	< 0.01	0.73
		None	High	7485*.*42	< 0.01	0.75
	Polarity	None	Low	4*.*00	< 0.01	0.00
		None	Medium	1813*.*12	< 0.01	0.18
		None	High	6349*.*25	< 0.01	0.63
	User Satisfaction	None	Low	2927*.*38	< 0.01	0.29
		None	Medium	1232*.*89	< 0.01	0.12
		None	High	4286*.*36	< 0.01	0.42

*Dist1* and *Dist2* refer to the distributions of the corresponding primary metric for the overall social network (after sharing 5 k posts) at the specified levels of tolerance and selective exposure as applicable based on the corresponding experiment (Exp). *H *statistic represents the Kruskal–Wallis test statistic. Effect size is computed using epsilon-squared (*ϵ*^2^)

**Table 5:** Statistical significance test results comparing a user’s POV (point-of-view) in the final population (after sharing 5 k posts) across different levels of selective exposure and user tolerance

Exp	Test	Dist1	Dist2	Test-Stat	*p* value	Effect size
Tolerant users	Paired *t* test	Low	Medium	1*.*35	= 0.18	0.02
		Medium	High	0*.*72	= 0.47	0.01
		High	Low	2*.*06	= 0.04	0.03
	One-way ANOVA	Low	Medium	1*.*08	= 0.30	0.02
		Medium	High	0*.*26	= 0.61	0.01
		High	Low	2*.*41	= 0.12	0.03
Selective exposure	Paired *t* test	None	Low	1*.*03	= 0.30	0.02
		None	Medium	10*.*20	< 0.01	0.17
		None	High	3*.*99	< 0.01	0.07
	One-way ANOVA	None	Low	0*.*56	= 0.45	0.02
		None	Medium	57*.*66	< 0.01	0.17
		None	High	9*.*48	< 0.01	0.07

*Dist1* and *Dist2* refer to the distributions of users’ POV (after sharing 5 k posts) at the specified levels of tolerance and selective exposure as applicable to the corresponding experiment (Exp). Effect size is computed using Cohen’s *d*

## Discussion

Polarization is slowed down substantially when tolerance in users is High. High tolerant users experience the least network polarization and have less network polarity than when users’ tolerance is Low. The Low polarization is plausibly because High tolerant users are less likely to change their stance on issues based on sanctions they receive than Low tolerant users, hence slowing down change to a user’s POV. The number of highly polarized users is the lowest when user tolerance is High. Our results are consistent with the earlier work (Coscia and Rossi 2022), which found lower levels of network polarization with high user tolerance in a social network.

Figure 1 shows user satisfaction when tolerance is Low is constantly higher than when tolerance is High, leading to higher overall user satisfaction. However, the number of users with positive satisfaction is higher when tolerance is High, compared to when tolerance is Medium or Low (Table 2). This indicates that the sharing of posts in a social network whose users have lower tolerance leads to higher overall user satisfaction but is concentrated among fewer users.

Surprisingly, High user tolerance leads to a more homophilic network (based on users’ POV) than when user tolerance is Low or Medium. Also, User reach (number of users who receive a post) is lower when tolerance in users is High compared to Low and Medium.

High selective exposure leads to higher polarization than Medium, Low, and None selective exposure, in that order. This is plausibly because when selective exposure is High, users are more likely to see congenial posts (posts that agree with their existing stance) and are subject to fewer posts that may challenge their stance. Our finding that higher selective exposure leads to higher polarization agrees with earlier findings from prior works (Stroud 2010; Garrett et al. 2014; Kim and Chen 2016). However, it is important to elucidate the difference in the methodology between our work and prior works to understand the results better. While ours is a multi-agent simulation that captures the evolution of polarization caused by the social interactions between users, prior works (Stroud 2010; Garrett et al. 2014; Kim and Chen 2016) primarily rely on self-reported survey data for their conclusions. Further, prior works focus on how exposure to some information may polarize an individual’s attitude in isolation rather than as a consequence of social interactions between multiple users.

As expected, user satisfaction is higher for higher levels of selective exposure (Figure 2). High user satisfaction may result because users receive more congenial posts with higher selective exposure, leading to more positive sanctions and higher user satisfaction for some users. The number of users with zero user satisfaction (i.e., users whose satisfaction didn’t change during the simulation run) is the highest when selective exposure is High and the number of negatively satisfied users is substantially lower (*≈* 2×) than lower levels of selective exposure. This indicates selective exposure ensures fewer users end up with aggregate negative satisfaction.

Higher selective exposure leads to the lowest user reach (i.e., the highest number of non-receivers, Table 1). This is most likely caused as a consequence of filtering out uncongenial posts for each user, which leads to fewer users receiving any given post than when no selective exposure is applied. The number of disinterested is the lowest in the case of High selective exposure demonstrating that selective exposure makes it less likely for a post to reach potentially disinterested (i.e., users with a potentially uncongenial POV toward the post). This comes at the cost of a low number of spreaders when selective exposure is High.

High selective exposure witnesses the least drop in highly active users between the start and the end of the simulation. Our findings on higher selective exposure leading to more highly active users are consistent with some empirical findings from prior work. Prior work (Stroud 2010) found selective exposure to congenial political information increases participation. At the same time, it undermines earlier work that found a positive role of cross-cutting exposure on political participation (Kim and Chen 2016).

High selective exposure leads to the highest number of highly polarized users at the end of the simulation. High selective exposure also leads to a social network with the highest homophily. Homophily shows some of the highest effect sizes in the statistical significance test analysis, with values indicating a very strong relation implying that the change in overall network homophily is statistically significant. The effect size is the highest when selective exposure is High, followed by Medium, and then Low, indicating an increasing pattern of homophily with higher selective exposure.

Our findings have practical and valuable implications for social networking platforms that have become an integral part of our lives. These platforms try to maximize user satisfaction and often employ content filtering (algorithmic selective exposure) to choose content based on user preference. Our simulation shows that achieving user satisfaction via selective exposure can potentially increase polarization in the social network. High selectivity in exposure to congenial content may lead to better user satisfaction (due to the increased likelihood of viewing congenial posts), but it also leads to more polarized users. On the other hand, social networks whose users have a higher tolerance experience far less polarization among their users for the same number of shared posts. However, user satisfaction when users’ tolerance is higher is lower.

Interestingly, network homophily (the tendency of being connected to users with similar POV) increases in both experiments, i.e., higher selective exposure and higher tolerance in users both lead to networks with higher homophily. Social networks with higher homophily are more prone to forming echo chambers (wherein a person encounters only beliefs or opinions that coincide with their own), which is a growing challenge for social media platforms. While it is not incumbent on social networking platforms to mitigate its ill effects, such as polarization among users and the formation of echo chambers, there are some benefits to it. For instance, our simulation shows higher selective exposure leads to the lowest user reach (i.e., the highest number of non-receivers).

Our simulation model is a step toward understanding the social interactions between users in a social network and how it influences user behavior and polarization. A better understanding of the potential consequences of the interactions on a social network can show us ways to mitigate the ill effects while still making the most of these social networking platforms.

### Threats to validity

Modeling user behavior is a challenging task that demands an intricate understanding of human psychology and an extensive operationalization of human traits. Though we model each user based on theories from social science and relevant observations from previous related works, the simplifications done to formalize the setup incur some threats to validity.

First, we assume equal strength of ties between each pair of connected users. In reality, people have varying strengths of ties, affecting how they react to posts from others and how it influences them.

Second, we only consider a user’s own preferences and content of the post when deciding to share a post, and providing sanctions. In reality, there may be a myriad of factors that affect such decisions.

Third, the simulation runs on artificially generated data. User attributes and the posts being shared are artificially generated based on suitable probability distributions. Though we ensured appropriate distributions for initial user attributes, this does not guarantee a reasonable replication of a real-world social network. Any generalizations based on these findings need to be verified with empirical data.

Forth, the results are based on simulation runs each of which ends after sharing ≈ 5000 posts. While most plots indicate the simulation stabilizing (near the end of the simulation) with the general direction of the plots being stable, there is no certainty that the same trends will continue forever.

The results should be taken with caution. Although our model is based on assumptions grounded in prior studies on polarization on social media, we use artificially generated data for this analysis. Further, reliably modeling user behavior is non-trivial and requires a fine-grained understanding of user behavior. We make simplifying assumptions in our model.

### Future directions

This work brings forth exciting directions for further research. First, it would help to develop richer simulation models that capture the dynamics of social networks, such as forming and severing ties between users and diffusing several posts simultaneously in the network. Second, it would help to seed the simulation with data collected from real users via a human-subject study. Third, it would be interesting to extend our model to incorporate methods of intervention that can help mitigate polarization in a social network.

## Data Availability

We have provided the link to the GitHub repository (https://github.com/ahaque2/MultiAgent-Social-Simulation.git) that contains the data, code, and results. The readme includes the instructions to reproduce the results. The link to the codebase (and initial seed data used) is mentioned at the end of section 3.2 and is publicly available.

## Appendix

See Tables 6 and 7.

**Table 6:** Notations used to describe the simulation design

Notation	Description
*c*_1_	A constant (scale factor) to scale up smaller values. We use the value of 10
*c*_2_	A constant (scale factor) to scale down the larger values. We use the value of 0.1
*a*_*x*_	Agent *x*
*p*_*k*_	*k*th post shared on the social network
*uS*(*a*_*x*_*, i, p*_*k*_)	Stance of *a*_*x*_ toward issue *i* after *p*_*k*_ is shared
*pS*(*p*_*k*_*, i*)	Stance of *p*_*k*_ toward issue *i*
*uA*(*a*_*x*_*, p*_*k*_)	Activity score for *a*_*x*_ after *p*_*k*_ is shared
*sPref* (*a*_*x*_*, p*_*k*_)	Sharing preference of *a*_*x*_ after *p*_*k*_ is shared
*sP* (*a*_*x*_*, p*_*k*_)	Probability of *a*_*x*_ to share *p*_*k*_
Sanc(*a*_*y*_*, p*_*k*_*, a*_*x*_)	Sanction *a*_*y*_ provides on receiving *p*_*k*_ from *a*_*x*_
*δS*(*a*_*x*_*, a*_*y*_*, i, p*_*k*_)	Difference in stance between the spreader (*a*_*x*_) and the receiver (*a*_*y*_) on issue *i* as post *p*_*k*_ is being shared
∆*S*(*a*_*x*_*, a*_*y*_*, i, p*_*k*_)	Shift in stance (of *a*_*x*_) due to a sanction (by *a*_*y*_) for *p*_*k*_ it shared on the issue *i*
POV (*a*_*x*_*, p*_*k*_)	POV (Point-of-View) of *a*_*x*_ after *p*_*k*_ has diffused in the social network
neigbhor(*G, a*_*x*_*, p*_*k*_)	All neighbors of *a*_*x*_ in the social network *G* which receive *p*_*k*_ from *a*_*x*_
numAgents(*G*)	Total number of agents in the social network *G*

**Table 7:** Secondary metrics to compare initial and final user distribution based on agent’s state

Metric	Description
Negative satisfied	Agents with user satisfaction less than zero
Zero satisfied	Agents with user satisfaction equal to zero
Positive satisfied	Agents with user satisfaction greater than zero
Low activity	Agents with user activity lower than or equal to 0.75
Medium activity	Agents with user activity between [0.75, 0.90]
High activity	Agents with user activity greater than or equal to 0.90
Low polarized	Agents with POV (Point-of-View) in [*− *0.1, 0.1]
High polarized	Agents with POV (Point-of-View) greater than 0.1 or lower than − 0.1

## Footnotes

1 https://github.com/ahaque2/MultiAgent-Social-Simulation.git

2 https://networkx.org/documentation/stable/reference/algorithms/assortativity.html
